# Immediate post-discharge care among US adults hospitalized with respiratory syncytial virus infection

**DOI:** 10.1186/s12890-024-03251-y

**Published:** 2024-10-04

**Authors:** Reiko Sato, Jen Judy, Kari Yacisin, Elizabeth Begier, Poorva Sardana, Neha Agrawal, Anchita Goswami, Manvi Sharma

**Affiliations:** 1 Value & Evidence, Pfizer Inc, Collegeville, PA 19426 USA; 2 Evidence Generation RWE team, Pfizer Inc, remote, MD USA; 3 Vaccines Medical Affairs, Pfizer Inc, Collegeville, PA USA; 4 Pfizer Vaccines, Dublin, Ireland; 5 Complete HEOR Solutions (CHEORS), 199 Folly Road, Chalfont, PA 18914 USA

**Keywords:** Respiratory Syncytial Virus, Respiratory infections, Adults, Hospitalization, Post-discharge care

## Abstract

**Background:**

Respiratory Syncytial Virus (RSV) is an important pathogen causing acute respiratory illnesses in adults. RSV infection can lead to severe outcomes, including hospitalizations and even death. Despite the increased recognition of the burden in older adults, immediate post-discharge care needs among adults hospitalized with RSV are not well characterized and have not been compared to other serious medical conditions (such as influenza, acute myocardial infarction (MI), and stroke) for which there have been long-standing disease prevention efforts.

**Objectives:**

This study aims to describe the immediate post-discharge care needs among adults hospitalized with RSV in the United States and descriptively compare it to those hospitalized with influenza, acute MI, or stroke.

**Design:**

Retrospective observational cohort study.

**Patients:**

Adults aged ≥ 18 years, hospitalized with a primary diagnosis of RSV, influenza, acute MI, or stroke from January 01, 2016, to December 31, 2019, were identified from the Premier Healthcare Database using the International Classification of Diseases (ICD-10) codes.

**Main measures:**

Immediate post-discharge care was categorized into three different levels of care based on the discharge dispositions. Descriptive analyses were performed.

**Key results:**

In total, 3,629 RSV, 303,577 influenza, 388,682 acute MI, and 416,750 stroke hospitalizations were identified, the majority occurred among patients aged ≥ 65 years. Professional home care needs were the highest for RSV hospitalizations (19.1%), followed by influenza (17.7%), stroke (15.4%), and acute MI (9.8%). Additionally, institutional care needs immediately following discharge were similar for RSV, influenza, and acute MI hospitalizations (14.2%, 15.8%, and 14.1%, respectively).

**Conclusions:**

Immediate post-discharge care needs among adults hospitalized with RSV, especially in older adults, can be considerable and comparable to influenza and acute MI discharges. With recently approved RSV vaccines, efforts to increase vaccination in older adults are needed to prevent RSV and associated healthcare consequences.

**Supplementary Information:**

The online version contains supplementary material available at 10.1186/s12890-024-03251-y.

## Introduction

Respiratory Syncytial Virus (RSV) is a common cause of acute respiratory illnesses in adults and usually causes mild, cold-like symptoms; however, RSV is increasingly recognized as a cause of serious illness among older adults, immunocompromised adults, and those with underlying medical conditions such as chronic heart and lung conditions [1–3]. Each year in the United States (US), RSV infection results in an estimated 60,000-160,000 hospitalizations, 119,000 emergency department admissions, 1.4 million outpatient visits, and 6,000–10,000 deaths among adults aged 65 years and older [4, 5]. RSV also imposes a substantial economic burden, with annual hospitalization costs in adults exceeding $1 billion in the US [6].

The burden of RSV in adults has remained largely underrecognized from the healthcare system, provider, and patient perspectives [4, 5, 7]. The limited awareness is due to multiple factors, including infrequent RSV testing, [8] lack of RSV-specific anti-viral treatments, [9] low sensitivity of standard-of-care clinical testing approaches, [10, 11] and no effective targeted prevention until the recent regulatory approval of RSV vaccines for older adults [12]. Attempts to raise awareness have been made by comparing the clinical, economic, and humanistic burden of RSV to a well-recognized viral illness, influenza, that causes a significant public health burden [13–18]. Previous studies have reported substantial care needs among adults hospitalized with influenza following discharge [13, 17]. Additionally, few studies reported similar or worse clinical outcomes in adults hospitalized with RSV than influenza [13, 14, 17, 19]. These studies indicated that follow-up care after RSV-associated hospitalization is common and extends well beyond the acute phase of the illness. However, these studies were limited by sample size and geography, which made it difficult to discern whether the post-discharge care was attributable to the RSV infection or other underlying conditions.

The clinical outcomes and the intensity of healthcare resource use associated with RSV hospitalization and post-discharge care have not been considered in the context of other common causes of hospitalization, such as acute myocardial infarction (MI) and stroke. These conditions are often targets for prevention by healthcare providers and healthcare systems due to their prevalence, high costs, availability of lifestyle and medication strategies for prevention and risk reduction, and the potential for additional care needs after hospitalization [20–24]. In this new era of RSV vaccine availability for older adults, [12] mobilization of primary prevention efforts should prioritize costly diseases that lead to hospitalization and higher-level care following discharge. We aim to further understand the medical care needs immediately after discharge from RSV hospitalizations and descriptively compare that to the care needs of other common acute medical events such as influenza, acute MI, or stroke hospitalizations.

## Methods

### Data source

This retrospective cohort study utilized data from the Premier Healthcare Database (PHD), which contains all-payer hospital administrative and billing data from a nationwide network of US hospitals captured across a geographically diverse area. Inpatient admissions include over 160 million visits, with more than 9 million per year since 2012, representing approximately 25% of annual US inpatient admissions [25]. This dataset includes information on demographics and disease states, admission and discharge diagnoses, data on billed services, patient dispositions, and discharge health status, with less than 0.01% missing data [25]. All data in PHD were structured, de-identified, and fully compliant with the Health Insurance Portability and Accountability Act of 1996 (HIPAA); [26, 27] The study did not require informed consent or institutional review board approval.

### Study population

The study population consisted of adults aged ≥ 18 years, hospitalized with a primary diagnosis of RSV, influenza, acute MI, or stroke between January 1, 2016, to December 31, 2019, defined as the assessment period. Data beyond 2019 were not included to limit the impact of the COVID-19 pandemic on changes in patient preferences for receiving medical services as well as changes in hospital discharge considerations [28, 29]. The four conditions were identified using the International Classification of Diseases, 10th revision, Clinical Modification (ICD-10-CM) codes (Table S1 in Supplemental Material 1). Hospitalizations were classified into four different cohorts (RSV, influenza, acute MI, and stroke). Because a patient could have multiple hospitalizations due to the four conditions of interest during the assessment period, we allowed patients to contribute to more than one disease cohort or the same disease cohort more than once if the additional hospitalization met the inclusion/exclusion criteria described below.

### Inclusion and exclusion criteria

The study included hospitalizations for one of the four conditions of interest (RSV, influenza, acute MI, or stroke), and primary diagnosis was used to determine the reason for hospitalization. Patients were admitted from a non-healthcare facility point of origin (i.e., community-dwelling individuals) and subsequently discharged or transferred to either home or self-care, home health organization, hospice-home, hospice-medical facility, another rehab facility, skilled nursing facility (SNF), intermediate care facility (ICF), long-term care (LTC) hospital, or other facilities, or those who died during their hospital stay. These discharge dispositions were the 10 most common and accounted for approximately 98% of discharge records. Hospitalizations in which patients were admitted from clinics or transferred from inpatient facilities, LTC facilities, or home health aides were excluded. Hospitalizations were also excluded if the patient had more than one diagnosis of conditions of interest (RSV, influenza, acute MI, or stroke) in the primary and secondary diagnosis positions during the same hospitalization. Lastly, readmissions due to the primary diagnosis of one of the four conditions within 90 days of any prior hospitalization related to those conditions were excluded. The included hospitalizations, referred to as qualifying hospitalizations, were analyzed separately for each of the four conditions of interest (Algorithm S1 and Figure S1 in Supplemental Material 1).

### Socio-demographic, clinical characteristics, and healthcare resource utilization

Demographics (age, sex, race), hospital geographic region, and payer type were captured. Risk conditions assessed included cardiopulmonary, cardiovascular, hematological, hepatic, metabolic, neurologic, pulmonary, and renal conditions, as well as immunocompromising and obesity-related comorbidities. These conditions were defined based on RSV and influenza risk factors outlined by the US Advisory Committee of Immunization Practices (ACIP) [30, 31] and were further identified using ICD-9-CM and ICD-10-CM codes (Supplemental Material 2). All comorbidities were assessed during each qualifying hospitalization as well as during a period of up to 12 months prior to the qualifying hospitalization at the same hospital facility. The qualifying hospitalizations were classified as high-risk if at least one primary or secondary diagnosis of the mentioned comorbidities was present. Charlson comorbidity index (CCI) was calculated to summarize morbidity. Assessment of healthcare resource utilization included intensive care unit (ICU) admission, mechanical ventilator use, inpatient length of stay (LOS), and length of ICU stay.

### Measured outcomes

The primary study outcome was immediate post-discharge care categorized into three levels as outlined in Table 1: elevated, moderate, and same or lower level of care. In-hospital death was also captured.

**Table 1:** Levels of immediate post-discharge care

Admitting source*	Discharge disposition category	Discharge disposition description†	Levels of care
Non-Healthcare Facility Point of Origin	Death	Expired	In-hospital death
	Rehabilitation Facility	Discharged/transferred to another rehab facility	Elevated care
	LTC Facility	Discharged/transferred to SNF	Elevated care
		Discharged/transferred to ICF	
		Discharged to hospice-medical facility	
		Discharged/transferred to a LTC hospital	
	Hospital or Inpatient Facility	Discharged/ transferred to other facility	Elevated care
	Home Health	Discharged to home health organization	Moderate care
		Discharged to hospice-home	
	Home or Self-Care	Discharged to home or self-care	Same or lower care

List of Abbreviations: ICF: Intermediate Care Facility; LTC: Long-term Care; SNF: Skilled Nursing Facility

Notes:

*Only “Non-Healthcare Facility Point of Origin” as admitting source was utilized

†Feasibility assessments were conducted. Top 10 discharge disposition codes were used for the analysis

### Statistical analysis

All analyses were conducted at the hospitalization level. Descriptive statistics were used to summarize socio-demographic, hospital and clinical characteristics, and healthcare resource utilization. Categorical variables were described using frequencies and percentages, while for continuous variables, mean and standard deviation were reported. To describe the immediate post-discharge care, frequencies, and percentages of discharge disposition categories, along with the defined levels of care (as outlined in Table 1) were reported. A Sankey diagram was employed to visually represent the transition in care. The results were separately summarized for each of the four cohorts. Analyses were also stratified by age (18–64 [younger adults] vs. ≥65 [older adults] years). Data management and analysis were conducted using SAS version 9.4 (SAS Institute, Cary, NC).

## Results

**Fig. 1:**
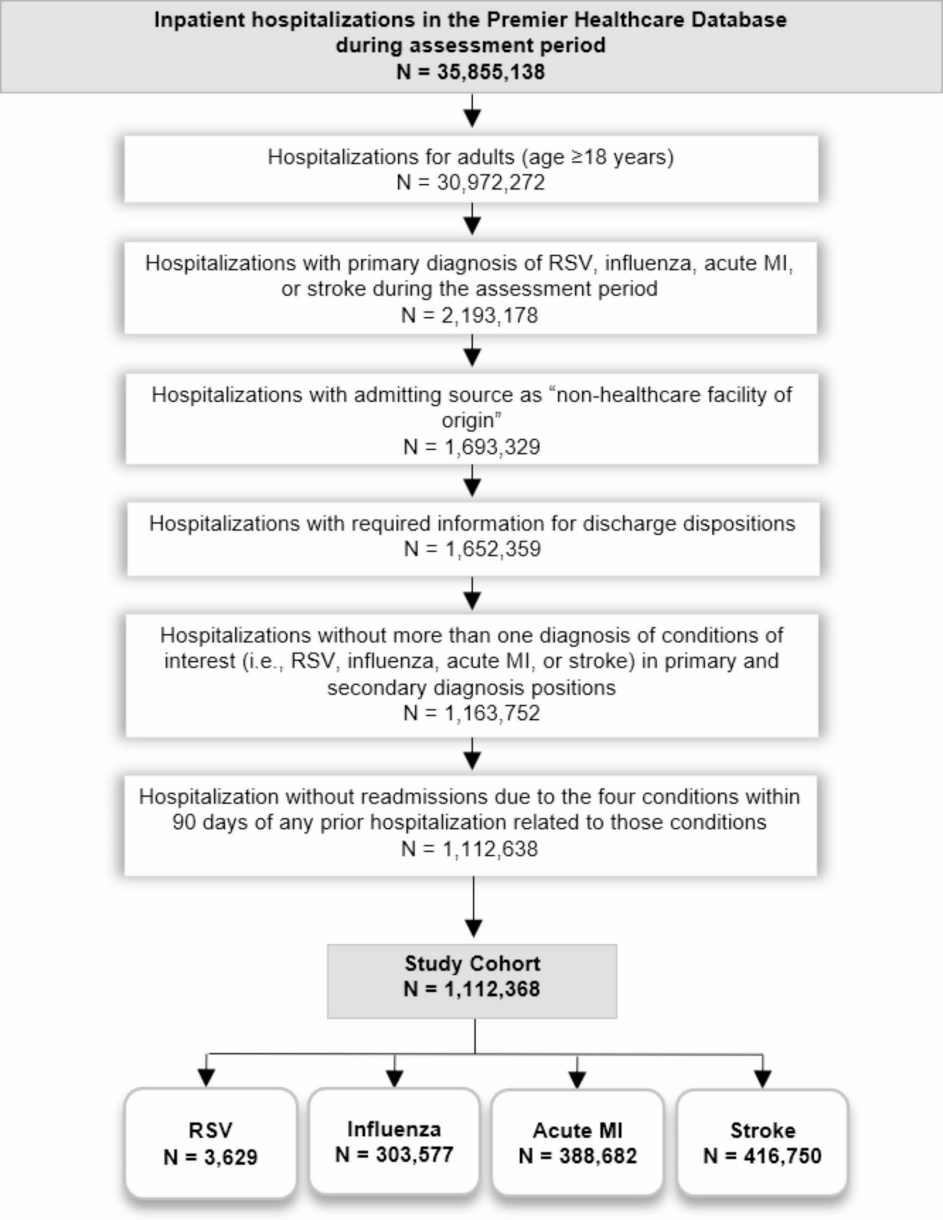
Flow diagram of study cohort List of Abbreviations: ICD: International Classification of Diseases; MI: Myocardial Infarction; N: Number of unique hospitalizations; RSV: Respiratory Syncytial Virus

Among 1,112,638 qualifying hospitalizations identified from the PHD during the assessment period (Fig. 1), there were 3,629 RSV, 303,577 influenza, 388,682 acute MI, and 416,750 stroke hospitalizations, with mean ages of 71.4, 65.5, 65.9, and 69.8 years, respectively (Table 2). A high proportion of hospitalizations were observed among older adults (≥ 65 years), ranging from 70.5% in RSV cohort to 53.6% in acute MI cohort. There were more females in RSV and influenza cohorts, more males in the acute MI cohort, and similar gender distribution in the stroke cohort (Table 2). Approximately half of the hospitalizations in RSV, influenza, and stroke cohorts had a cardiovascular comorbidity. In the acute MI cohort, all hospitalizations with acute MI at presentation were counted towards the pre-existing cardiovascular comorbid condition frequencies (Table 3). Pulmonary comorbidities were common in RSV (43.8%) and influenza (71.2%) cohorts. Most qualifying hospitalizations (> 90%) included a diagnosis of a high-risk condition.

**Table 2:** Socio-demographic characteristics for adults hospitalized with RSV, influenza, acute MI, or stroke

Characteristics	RSV [*N* = 3,629]	Influenza [*N* = 303,577]	Acute MI [*N* = 388,682]	Stroke [*N* = 416,750]
Age (in years) [Mean (SD)]	71.4 (15.7)	65.5 (16.4)	65.9 (13.6)	69.8 (13.9)
Age group (in years) [N (%)]				
18–64	1,070 (29.5)	128,751 (42.4)	180,234 (46.4)	142,648 (34.2)
≥ 65	2,559 (70.5)	174,826 (57.6)	208,448 (53.6)	274,102 (65.8)
Sex [N (%)]				
Male	1,312 (36.2)	135,696 (44.7)	244,451 (62.9)	207,683 (49.8)
Female	2,317 (63.8)	167,838 (55.3)	144,162 (37.1)	208,981 (50.1)
Unknown	-	43 (0.0)	69 (0.0)	86 (0.0)
Race [N (%)]				
White	2,707 (74.6)	229,437(75.6)	299,314 (77.0)	297,034 (71.3)
Black	433 (11.9)	45,023 (14.8)	44,563 (11.5)	72,715 (17.4)
Asian	97 (2.7)	5,339 (1.8)	9,076 (2.3)	11,191 (2.7)
Other	305 (8.4)	19,300 (6.4)	28,447 (7.3)	28,235 (6.8)
Unable to determine	87 (2.4)	4,478 (1.5)	7,282 (1.9)	7,575 (1.8)
Hospital Geographic Region [N (%)]				
Midwest	684 (18.8)	67,826 (22.3)	78,769 (20.3)	79,628 (19.1)
Northeast	1,204 (33.2)	45,770 (15.1)	59,551 (15.3)	63,341 (15.2)
South	1,420 (39.1)	137,955 (45.4)	185,620 (47.8)	207,639 (49.8)
West	321 (8.8)	52,026 (17.1)	64,742 (16.7)	66,142 (15.9)
Payer Type [N (%)]				
Medicare	2,690 (74.1)	198,275 (65.3)	215,123 (55.3)	276,971 (66.5)
Medicaid	321 (8.8)	37,994 (12.5)	34,987 (9.0)	36,018 (8.6)
Commercial	505 (13.9)	47,385 (15.6)	104,018 (26.8)	74,375 (17.8)
Uninsured	61 (1.7)	11,924 (3.9)	22,340 (5.7)	19,266 (4.6)
Others	52 (1.4)	7,999 (2.6)	12,214 (3.1)	10,120 (2.4)

List of Abbreviations: MI: Myocardial Infarction; N: Number of unique hospitalizations; RSV: Respiratory Syncytial Virus

**Table 3:** Clinical characteristics and healthcare resource utilization for adults hospitalized with RSV, influenza, acute MI, or stroke

Characteristics	RSV [*N* = 3,629]	Influenza [*N* = 303,577]	Acute MI [*N* = 388,682]	Stroke [*N* = 416,750]
CCI [Mean (SD)]	2.9 (2.4)	3.0 (2.5)	3.1 (2.2)	3.8 (2.3)
Risk conditions [N (%)]				
Cardiopulmonary	198 (5.5)	21,279 (7.0)	11,574 (3.0)	8,492 (2.0)
Cardiovascular	2,030 (55.9)	166,549 (54.9)	388,682 (100.0)	203,549 (48.8)
Hematological	638 (17.6)	41,996 (13.8)	30,756 (7.9)	27,249 (6.5)
Hepatic	104 (2.9)	12,946 (4.3)	6,169 (1.6)	6,909 (1.7)
Metabolic	1,349 (37.2)	108,623 (35.8)	157,666 (40.6)	167,403 (40.2)
Neurologic	897 (24.7)	67,252 (22.2)	103,024 (26.5)	377,531 (90.6)
Pulmonary	1,591 (43.8)	216,071 (71.2)	82,984 (21.4)	79,340 (19.0)
Renal	1,067 (29.4)	74,321 (24.5)	87,775 (22.6)	81,971 (19.7)
Immunocompromising	621 (17.1)	45,778 (15.1)	22,807 (5.9)	30,531 (7.3)
Obesity-related	424 (11.7)	45,680 (15.0)	34,589 (8.9)	28,501 (6.8)
Risk Group [N (%)]				
High-risk	3,311 (91.2)	282,039 (92.9)	388,682 (100.0)	402,259 (96.5)
Healthcare Resource Utilization				
ICU admission [N (%)]	111 (3.1)	58,402 (19.2)	106,291 (27.3)	93,638 (22.5)
MV Use [N (%)]	19 (0.5)	48,098 (15.8)	23,112 (5.9)	6,527 (1.6)
Length of inpatient stay [Mean (SD)]	4.0 (2.8)	4.3 (4.4)	3.3 (3.1)	4.2 (4.8)
Length of ICU stay [Mean (SD)]	2.3 (2.3)	2.8 (3.8)	1.5 (1.6)	1.9 (2.4)

List of Abbreviations: CCI: Charlson Comorbidity Index; ICU: Intensive Care Unit; MI: Myocardial Infarction; MV: Mechanical Ventilator; N: Number of unique hospitalizations; RSV: Respiratory Syncytial Virus

The proportion of ICU admissions was highest in the acute MI cohort (27.3%) and lowest in the RSV cohort (3.1%). The highest proportion of mechanical ventilator use was reported for influenza hospitalizations (15.8%), followed by acute MI (5.9%), stroke (1.6%), and RSV (0.5%) hospitalizations. The mean inpatient LOS was similar for RSV, influenza, acute MI, and stroke cohorts, ranging from 3.3 to 4.3 days. The mean ICU LOS ranged from 1.5 to 2.8 days.

**Fig. 2:**
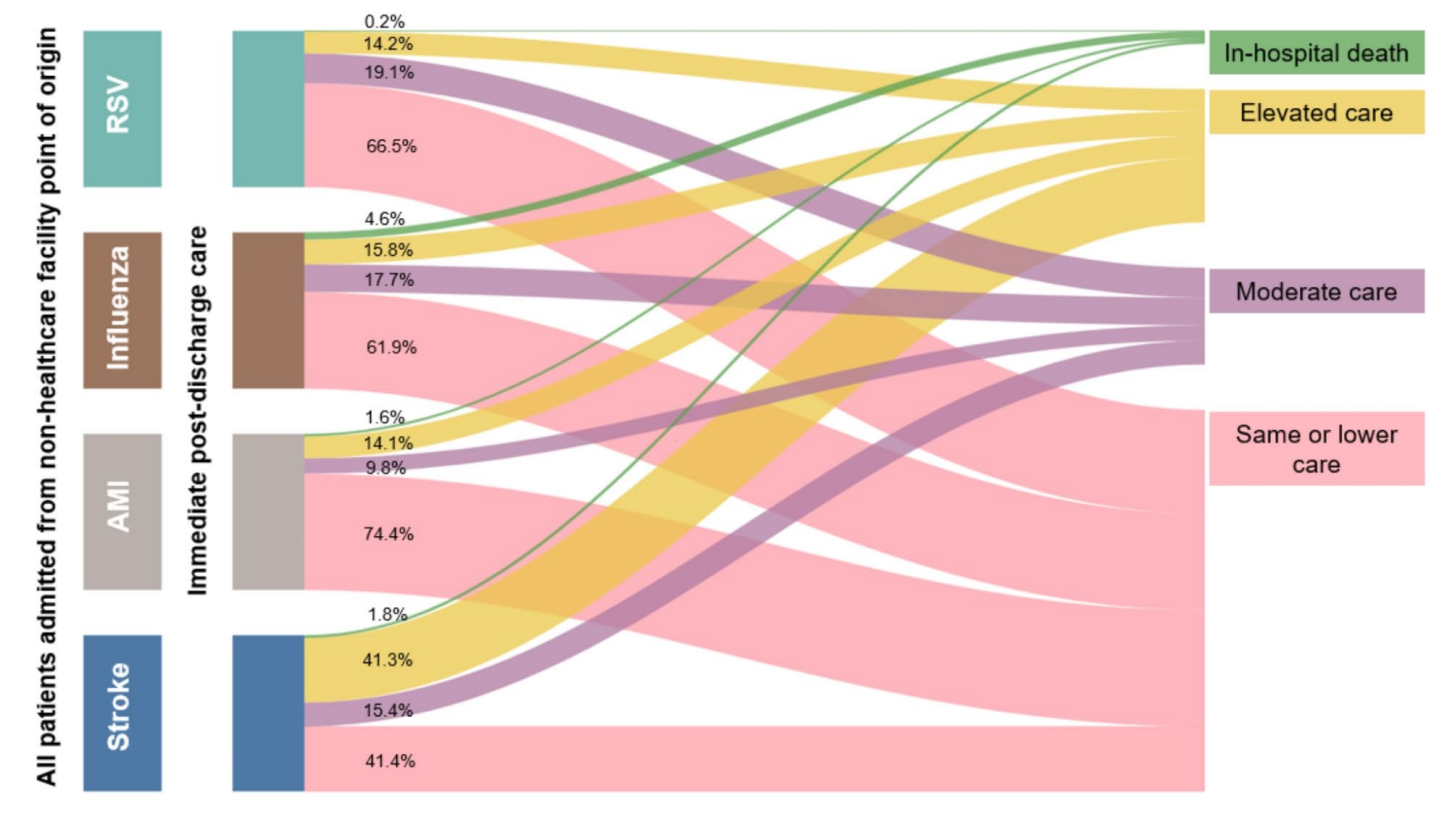
Immediate post-discharge care or in-hospital death among adults hospitalized with RSV, influenza, acute MI, or stroke List of Abbreviations: MI: Myocardial Infarction; RSV: Respiratory Syncytial Virus

Most hospitalizations for RSV, influenza, or acute MI were associated with patients being discharged to home or self-care (61.9–74.4%), with the exception of the stroke cohort (41.4%) (Fig. 2 and Table S2 in Supplemental Material 1). Moderate care needs were 19.1% in the RSV cohort, similar to influenza (17.7%) and the stroke cohort (15.4%). Acute MI cohort had the lowest (9.8%) moderate care needs. Elevated care immediately after hospital discharge was 14.2% for RSV, similar to influenza (15.8%) and acute MI (14.1%). A notable proportion of the stroke cohort needed elevated care (41.3%).

In age-stratified analysis, the mean ages ranged from 50.2 to 54.1 years for younger adults and 76.3 to 79.8 years for older adults (Table S3 in Supplemental Material 1). The hospital LOS between younger and older adults was similar across the four cohorts. Most younger adults also had high-risk conditions similar to the older adults across the four cohorts. Interestingly, ICU admissions were similar or slightly higher in the younger than older cohorts across all four conditions (Table S4 in Supplemental Material 1). In general, older adults needed greater levels of both elevated and moderate care as compared to younger adults across all four cohorts (Table 4). Among older adults, the percentage with elevated level of care needs was similar for RSV (18.8%), influenza (21.4%), and acute MI (19.3%), but not stroke (48.1%). A need for a moderate level of post-hospitalization care was also prevalent among older adults, ranging from 14.1% in the acute MI cohort to 23.9% in the RSV cohort. A noteworthy proportion of younger adults also needed either elevated or moderate care immediately after discharge: 11.0%, 18.9%, 13.0%, and 39.7% of RSV, influenza, acute MI, and stroke hospitalizations, respectively.

**Table 4:** Immediate post-discharge care stratified by age group (in years) among adults hospitalized with RSV, influenza, acute MI, or stroke

Cohorts		RSV		Influenza		Acute MI		Stroke	
Levels of care* [*N* (%)]	Discharge dispositions [*N* (%)]	18–64 [*N* = 1,070]	≥ 65 [*N* = 2,559]	18–64 [*N* = 128,751]	≥ 65 [*N* = 174,826]	18–64 [*N* = 180,234]	≥ 65 [*N* = 208,448]	18–64 [*N* = 142,648]	≥ 65 [*N* = 274,102]
In-hospital death	Death	1 (0.1)	8 (0.3)	4,275 (3.3)	9,590 (5.5)	1,154 (0.6)	5,233 (2.5)	1,165 (0.8)	6,426 (2.3)
Elevated care	*Rehabilitation Facility*	*3* *(0.3)*	*36* *(1.4)*	*936* *(0.7)*	*2*,*655* *(1.5)*	*578* *(0.3)*	*2*,*714 (1.3)*	*24*,*570 (17.2)*	*53*,*732 (19.6)*
	*LTC Facility*	*26* *(2.4)*	*437* *(17.1)*	*7*,*054* *(5.5)*	*32*,*378 (18.5)*	*2*,*198* *(1.2)*	*21*,*387 (10.3)*	*11*,*757 (8.2)*	*73*,*011 (26.6)*
	*Hospital or Inpatient Facility*	*6* *(0.6)*	*7* *(0.3)*	*2*,*645* *(2.1)*	*2*,*392* *(1.4)*	*11*,*795* *(6.5)*	*16*,*117 (7.7)*	*4*,*041 (2.8)*	*5*,*020* *(1.8)*
	Total	35 (3.3)	480 (18.8)	10,635 (8.3)	37,425 (21.4)	14,571 (8.1)	40,218 (19.3)	40,368 (28.3)	131,763 (48.1)
Moderate care	Home Health	82 (7.7)	611 (23.9)	13,599 (10.6)	40,095 (22.9)	8,812 (4.9)	29,408 (14.1)	16,269 (11.4)	48,056 (17.5)
Same or lower care	Home or Self-Care	952 (89.0)	1,460 (57.1)	100,242 (77.9)	87,716 (50.2)	155,697 (86.4)	133,589 (64.1)	84,846 (59.5)	87,857 (32.1)

List of Abbreviations: LTC: Long-term Care; MI: Myocardial Infarction; N: Number of unique hospitalizations; RSV: Respiratory Syncytial Virus

*Only “Non-Healthcare Facility Point of Origin” as admitting source was considered

## Discussion

Our study found that approximately one-third of RSV hospitalizations resulted in institutional and professional home care needs immediately following discharge and were comparable to care needs of adults hospitalized with influenza or acute MI. RSV hospitalizations had similar durations of inpatient and ICU stays when compared with influenza, acute MI, and stroke hospitalizations. Consistent with the age-related trends for RSV-associated hospitalizations in the US [32] and other prior studies, [1, 4, 17, 19, 33] the present study found that a high percentage of RSV hospitalizations were among older adults (> 70%) and adults with high-risk conditions (> 90%). Further, our study found that a notable proportion (11%) of younger adults with RSV also required a higher level of care post-discharge than prior to admission. Among these younger adults, about 90% had at least one of the underlying risk conditions, demonstrating that even younger adults with underlying risk conditions required additional healthcare resources after RSV hospitalization discharge.

Few studies have documented the post-discharge care needs for adults hospitalized with RSV. A chart-review study [34] reported that 76% of older adults and 73% of other adults without chronic lung disease hospitalized with RSV required follow-up care after discharge, indicating a substantial post-discharge burden is present across different age groups. In a prospective global study that included adults ≥ 18 years who were hospitalized with RSV, 24.5% required professional home care, and 11.6% required institutional care after discharge [13]. In a retrospective study of US adults hospitalized with RSV, 10–16% required skilled nursing either at home or at a nursing facility post-discharge compared with 6.7% before the RSV admissions [34]. Among the subgroups examined, older adults and immunocompromised adults most frequently required skilled nursing [34]. This is consistent with our study, where older adults discharged from RSV hospitalization entered institutional care and home health programs at a much higher proportion than younger adults. However, in contrast to our study, many of these previous studies had limited sample sizes, and some did not indicate whether the higher-level care needs were immediately following discharge or within a defined period post-discharge.

The higher-level care needs after RSV hospitalization may partially be explained by the acute functional decline associated with RSV hospitalization in older adults [35]. In a longitudinal US study, older adults who were living in a community with assistance prior to admission had a significant decline in instrumental activities of daily living at 6 months after RSV hospitalization, while there were no significant changes in older adults living independently in the community [35]. This highlights that even among community-dwelling individuals, certain subgroups are at higher risk for functional decline from RSV hospitalization and the ensuing loss of independence that contributes to the need for elevated care after discharge.

The present study found that the needs for professional home health or institutional care immediately after discharge were comparable between the RSV and influenza cohorts. Additionally, hospitalizations with RSV exhibited a similar mean length of hospital and ICU stay as hospitalizations with influenza. This corroborates findings from prior studies which reported that the morbidity, mortality, and medical resource utilization of RSV hospitalizations were of similar magnitude as influenza hospitalizations [13, 14]. A previous study by Pastula et al. 2017, also reported a higher mean LOS (6 days) for RSV hospitalizations as compared to influenza hospitalizations (3.6 days) [15]. Similar trends were also seen in other countries [17, 18]. Unlike a few other published studies, [13, 14, 17, 36] our study found lower ICU admissions and mechanical ventilator use in patients hospitalized with RSV as compared to those with influenza. This is likely due to the higher proportion of underlying pulmonary conditions in the influenza cohort (71.2%) than in the RSV cohort (43.8%). Furthermore, lower utilization of high-intensity care (such as ICU admission and mechanical ventilator use) despite similar lengths of hospital and ICU stays in the RSV cohort as compared to the influenza cohort suggests that there may be differences in the type of resources used in the hospital across the two cohorts depending on underlying risk conditions. Given how infrequent RSV testing is in the US, this finding may not be representative of all patients with RSV infection [8].

Our study is unique in that we descriptively compared healthcare utilization and post-discharge care of RSV to completely different but important medical events of acute MI and stroke. Healthcare providers and payer organizations have focused on these common but serious medical conditions because effective preventive interventions are available. There have been substantial investments and awareness towards disease prevention efforts for influenza, acute MI, and stroke [20, 37]. By documenting that healthcare resource utilization and overall immediate post-discharge care needs for adults hospitalized with RSV are similar to needs of adults hospitalized with influenza, acute MI, or stroke, this study underscores the need for prioritizing RSV prevention efforts among older adults similar to influenza, acute MI, and stroke. With the recent regulatory approvals and US Advisory Committee on Immunization Practices (ACIP) recommendations of RSV vaccines for adults aged ≥ 75 years and those 60 to 74 years with risk conditions, [12, 38, 39] efficient implementation and high uptake among older adults will be important for RSV prevention [31].

Several limitations of this study should be noted. The PHD is an administrative and billing data set, not collected for research purposes; therefore, there may be reporting errors and omissions, thereby leading to potential misclassification. Although the admitting source was limited to a non-healthcare facility point of origin, mostly involving community-dwelling individuals, the extent of patients’ specific health and functional assistance needs in that setting prior to the hospitalization is unknown. Despite data contribution from over 1,190 hospitals across the US, [25] the patients hospitalized with RSV, influenza, acute MI, or stroke in the current study may not be representative of all US adults. In particular, RSV testing is not generally routinely conducted in adults presenting to the hospital with acute respiratory symptoms [8]. Therefore, the RSV cohort is an undercount of RSV-associated hospitalizations and may not be representative of the full clinical spectrum of adults hospitalized with RSV. This study only considered primary hospital diagnosis for identification of hospitalizations associated with conditions of interest. In the case of RSV, it is possible that hospitals use a different code as the primary diagnosis if an underlying condition is exacerbated that requires serious management, further undercounting patients with RSV and potentially impacting the representativeness of this cohort.

This study required a 90-day clean post-discharge period (i.e., no subsequent hospitalization associated with either of the four conditions of interest within 90 days of their previous hospitalizations associated with any of the four conditions of interest) to identify qualifying hospitalizations for inclusion. Readmissions within a short window due to the four conditions were not included and it is likely that there is additional higher-level care after discharge from the readmission that is not captured in the current study. Furthermore, since this study descriptively summarized outcomes associated with RSV hospitalizations to influenza, acute MI, and stroke hospitalizations using summary measures, no statistical inference testing was done across the four cohorts or in a pairwise fashion. When characterizing cardiovascular comorbidities, all acute MI hospitalizations were identified as having these and given that acute MI can occasionally result from non-cardiovascular factors, [40] it is possible that not all patients hospitalized with acute MI had cardiovascular comorbidities. This potential misclassification is not likely to impact the results presented in this study. Despite these limitations, the study includes information on a large number of patients across demographic profiles, payers, and diverse US geographic regions that improved prior study limitations on care immediately after discharge.

In conclusion, this study documents that immediate post-discharge care needs among adults hospitalized with RSV infection are considerable, especially among older adults. Adults discharged from RSV hospitalization had similar or higher use of professional home care as compared to adults discharged from influenza, acute MI, or stroke hospitalization. Elevated care involving institutional care immediately after discharge from RSV hospitalization was common with similar frequency as discharge from influenza or acute MI hospitalization. Given the substantial care needs following RSV hospitalizations, efforts to implement preventive strategies, including RSV vaccination in older adults, are needed to reduce the continued healthcare burden after RSV hospitalization.

## Electronic supplementary material

Below is the link to the electronic supplementary material.

Supplementary Material 1

Supplementary Material 2

## Data Availability

The proprietary data supporting the results of this study is owned by PINC AI™ Applied Sciences but restrictions apply to the availability of these data, which were used under license for the current study, and so are not publicly available. Interested researchers may contact solutioncenter@premierinc.com for data access requests.

## Declarations

### Clinical trial number

Clinical trial number is not applicable as this study was a retrospective observational study.
