# Supplementary material for: Immediate post-discharge care among US adults hospitalized with respiratory syncytial virus infection
Source: BMC Pulm Med. 2024 Oct 4;24:486. doi: 10.1186/s12890-024-03251-y (PMC11451113; doi:10.1186/s12890-024-03251-y)
Supplement: Supplementary file 1 — Supplementary Material 1 [file 12890_2024_3251_MOESM1_ESM.docx]

**Immediate Post-Discharge Care among US Adults Hospitalized with Respiratory Syncytial Virus Infection**

**Supplemental Material 1**

**Contents**

[**Algorithm S1:** Stepwise process for identifying the qualifying hospitalizations associated with the conditions of interest 2](#_Toc154754573)

# **List of Figures**

[**Figure S1:** Classification approach for multiple hospitalizations of a given patient on distinct dates 3](#_Toc154753667)

# **List of Tables**

[**Table S1:** ICD-10 CM codes for conditions of interest 5](#_Toc154755637)

[**Table S2:** Immediate post-discharge care among adults hospitalized with RSV, influenza, acute MI, and stroke 5](#_Toc154755638)

[**Table S3:** Socio-demographic characteristics for adults hospitalized with RSV, influenza, acute MI, and stroke stratified by age group 6](#_Toc154755639)

[**Table S4:** Clinical characteristics and healthcare resource utilization for adults hospitalized with RSV, influenza, acute MI, and stroke stratified by age group 7](#_Toc154755640)

# **Algorithm S1: Stepwise process for identifying the qualifying hospitalizations associated with the conditions of interest**

The steps mentioned below describe the process of identifying qualifying hospitalizations, when a patient had multiple hospitalizations due to any of the four conditions during the assessment period.

1. For each hospitalization for the four conditions identified during the assessment period (using primary diagnosis position), a 90-day post-discharge period window was created.
2. The earliest hospitalization in the assessment period was defined as the index hospitalization for that condition (e.g., if the earliest of all hospitalizations was associated with RSV, it was termed as RSV_1_) after ensuring that there were no hospitalizations associated with the four conditions of interest within 90-days before that hospitalization (i.e., 90-day clean baseline).

Note: The earliest hospitalization in the assessment period was excluded from the analysis if there existed other hospitalization(s) associated with the four conditions of interest 90 days before that hospitalization until a hospitalization for conditions of interest was selected such that there exists a 90-day clean baseline. The selected hospitalization was referred to as the first cohort-specific index hospitalization.

1. Any subsequent hospitalization associated with either of the four conditions of interest within 90-day post-discharge period of their previous hospitalizations associated with any of the four conditions of interest (if present) was not considered as a separate hospitalization and thereby, not used for outcome assessment.
2. The earliest hospitalization for any of the four conditions (in primary diagnosis position) beyond the 90-day clean post-discharge period of all previous hospitalizations (if present) was considered as separate hospitalization.
3. All subsequent hospitalizations due to primary diagnosis of any of the four conditions (if present) were defined in the similar manner using the 90-day clean post-discharge period criteria from all previous hospitalizations.

Figure S1 summarizes the approach used for the classification of multiple hospitalizations for a given patient.

**Figure S1: Classification approach for multiple hospitalizations of a given patient on distinct dates**


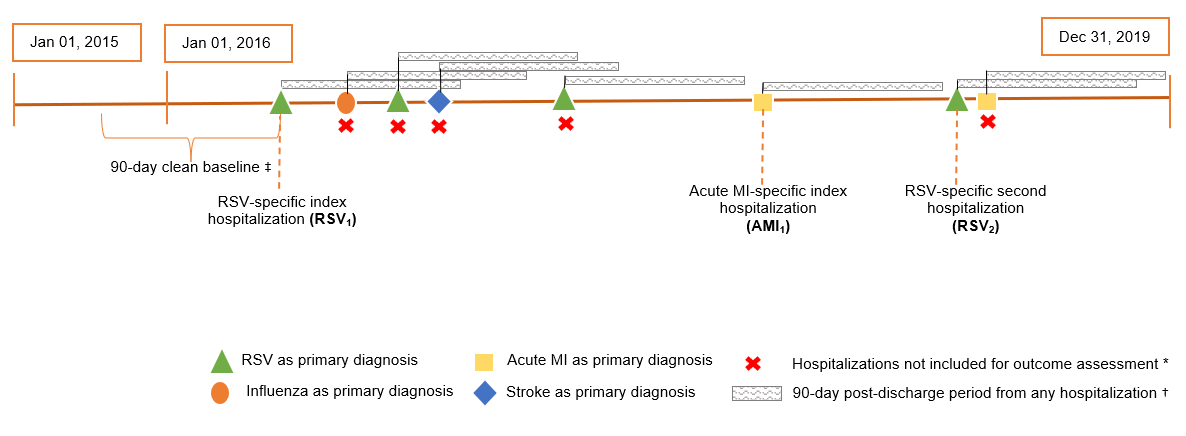


List of Abbreviations: AMI: Acute Myocardial Infarction; MI: Myocardial Infarction; RSV: Respiratory Syncytial Virus.

Notes:

* For patients with multiple hospitalizations during the study period, we ensured at least a 90-day period between hospitalization discharge date and the next admission date for hospitalization due to either of the four conditions of interest (RSV, influenza, acute MI, or stroke) for the hospitalization to be counted in one of the four cohorts.

† Multiple hospitalizations within the 90-day post-discharge period related to any of the four conditions of interest were not counted as qualifying hospitalizations.

‡ A 90-day clean baseline (i.e., no hospitalizations associated with the four conditions of interest within 90-day before that hospitalization) was applied.

**Table S1: ICD-10 CM codes for conditions of interest**

| **Conditions** | **ICD-10 CM Code** |
| --- | --- |
| RSV | B97.4, J20.5, J12.1, J21.0 |
| Influenza | J9, J10, J11 |
| Acute MI | I21, I22 |
| Stroke | I60, I61, I62, I63 |

List of Abbreviations: ICD: International Classification of Disease; MI: Myocardial Infarction; RSV: Respiratory Syncytial Virus.

**Table S2: Immediate post-discharge care among adults hospitalized with RSV, influenza, acute MI, and stroke**

| **Level of care***  **[N (%)]** | **Discharge dispositions [N (%)]** | **RSV**  **[N=3,629]** | **Influenza**  **[N=303,577]** | **Acute MI**  **[N=388,682]** | **Stroke**  **[N=416,750]** |
| --- | --- | --- | --- | --- | --- |
| Death | Inpatient death | 9 (0.2) | 13,865 (4.6) | 6,387 (1.6) | 7,591 (1.8) |
| Elevated care | *Rehabilitation Facility* | *39 (1.1)* | *3,591 (1.2)* | *3,292 (0.8)* | *78,302 (18.8)* |
|  | *LTC Facility* | *463 (12.8)* | *39,432 (13.0)* | *23,585 (6.1)* | *84,768 (20.3)* |
|  | *Hospital or Inpatient Facility* | *13 (0.4)* | *5,037 (1.7)* | *27,912 (7.2)* | *9,061 (2.2)* |
|  | Total | 515 (14.2) | 48,060 (15.8) | 54,789 (14.1) | 172,131 (41.3) |
| Moderate care | Home Health | 693 (19.1) | 53,694 (17.7) | 38,220 (9.8) | 64,325 (15.4) |
| Same or lower care | Home or Self-Care | 2,412 (66.5) | 187,958 (61.9) | 289,286 (74.4) | 172,703 (41.4) |

List of Abbreviations: LTC: Long-term Care; MI: Myocardial Infarction; N: Number of unique hospitalizations; RSV: Respiratory Syncytial Virus.

*Only “Non-Healthcare Facility Point of Origin” as admitting source was utilized.

**Table S3: Socio-demographic characteristics for adults hospitalized with RSV, influenza, acute MI, and stroke stratified by age group**

| **Characteristics** | **RSV** | | **Influenza** | | **Acute MI** | | **Stroke** | |
| --- | --- | --- | --- | --- | --- | --- | --- | --- |
|  | **18-64**  **[N = 1,070]** | **≥ 65**  **[N = 2,559]** | **18-64**  **[N = 128,751]** | **≥ 65**  **[N = 174,826]** | **18-64**  **[N = 180,234]** | **≥ 65**  **[N = 208,448]** | **18-64**  **[N = 142,648]** | **≥ 65**  **[N = 274,102]** |
| Age (in years) [Mean (SD)] | 51.3 (10.8) | 79.8 (7.7) | 50.2 (12.3) | 76.8 (7.5) | 53.9 (7.8) | 76.3 (7.7) | 54.1 (8.5) | 78.0 (7.7) |
| Age group (in years) [N (%)] | | | | | | | | |
| 18-49 | 367  (34.3) | - | 46,027 (35.7) | - | 46,471 (25.8) | - | 34,875  (24.4) | - |
| 50-64 | 703  (65.7) | - | 82,724 (64.3) | - | 133,763 (74.2) | - | 107,773 (75.6) | - |
| 65-74 | - | 719  (28.1) | - | 75,511 (43.2) | - | 97,505  (46.8) | - | 102,441 (37.4) |
| 75-84 | - | 913  (35.7) | - | 62,542 (35.8) | - | 69,683  (33.4) | - | 98,092 (35.8) |
| ≥ 85 | - | 927  (36.2) | - | 36,773 (21.0) | - | 41,260  (19.8) | - | 73,569 (26.8) |
| Sex [N (%)] | | | | | | | | |
| Male | 406 (37.9) | 906 (35.4) | 59,647 (46.3) | 76,049 (43.5) | 126,071 (69.9) | 118,380 (56.8) | 82,227 (57.6) | 125,456 (45.8) |
| Female | 664 (62.1) | 1,653 (64.6) | 69,079 (53.7) | 98,759 (56.5) | 54,121  (30.0) | 90,041 (43.2) | 60,390 (42.3) | 148,591 (54.2) |
| Unknown | - | - | 25  (0.0) | 18  (0.0) | 42  (0.0) | 27  (0.0) | 31  (0.0) | 55  (0.0) |
| Race [N (%)] | | | | | | | | |
| White | 677 (63.3) | 2,030 (79.3) | 87,855 (68.2) | 141,582 (81.0) | 130,862 (72.6) | 168,452 (80.8) | 86,406 (60.6) | 210,628 (76.8) |
| Black | 235 (22.0) | 198  (7.7) | 26,705 (20.7) | 18,318 (10.5) | 25,806 (14.3) | 18,757  (9.0) | 36,911 (25.9) | 35,804 (13.1) |
| Asian | 25 (2.3) | 72  (2.8) | 1,837 (1.4) | 3,502 (2.0) | 4,024 (2.2) | 5,052 (2.4) | 3,787 (2.7) | 7,404 (2.7) |
| Other | 102  (9.5) | 203  (7.9) | 10,032 (7.8) | 9,268  (5.3) | 15,511  (8.6) | 12,936  (6.2) | 12,407 (8.7) | 15,828  (5.8) |
| Unable to determine | 31 (2.9) | 56  (2.2) | 2,322 (1.8) | 2,156 (1.2) | 4,031 (2.2) | 3,251 (1.6) | 3,137  (2.2) | 4,438 (1.6) |
| Hospital Geographic Region [N (%)] | | | | | | | | |
| Midwest | 211  (19.7) | 473  (18.5) | 29,480 (22.9) | 38,346 (21.9) | 36,903  (20.5) | 41,866  (20.1) | 26,531 (18.6) | 53,097 (19.4) |
| Northeast | 312  (29.2) | 892  (34.9) | 18,313 (14.2) | 27,457 (15.7) | 25,770  (14.3) | 33,781  (16.2) | 20,142 (14.1) | 43,199 (15.8) |
| South | 449  (42.0) | 971  (37.9) | 59,615 (46.3) | 78,340 (44.8) | 89,067  (49.4) | 96,553  (46.3) | 74,500 (52.2) | 133,139 (48.6) |
| West | 98  (9.2) | 223  (8.7) | 21,343 (16.6) | 30,683 (17.6) | 28,494  (15.8) | 36,248  (17.4) | 21,475 (15.1) | 44,667 (16.3) |
| Payer Type [N (%)] | | | | | | | | |
| Medicare | 325 (30.4) | 2,365 (92.4) | 37,472  (29.1) | 160,803 (92.0) | 27,926  (15.5) | 187,197  (89.8) | 26,201 (18.4) | 250,770 (91.5) |
| Medicaid | 252 (23.6) | 69  (2.7) | 35,585  (27.6) | 2,409  (1.4) | 32,257 (17.9) | 2,730  (1.3) | 32,022 (22.4) | 3,996  (1.5) |
| Commercial | 408 (38.1) | 97  (3.8) | 39,810  (30.9) | 7,575  (4.3) | 90,886  (50.4) | 13,132  (6.3) | 61,002 (42.8) | 13,373  (4.9) |
| Uninsured | 53  (5.0) | 8  (0.3) | 11,264  (8.7) | 660  (0.4) | 21,089  (11.7) | 1,251  (0.6) | 17,600 (12.3) | 1,666  (0.6) |
| Others | 32  (3.0) | 20  (0.8) | 4,620  (3.6) | 3,379  (1.9) | 8,076  (4.5) | 4,138  (2.0) | 5,823  (4.1) | 4,297  (1.6) |

List of Abbreviations: MI: Myocardial Infarction; N: Number of unique hospitalizations; RSV: Respiratory Syncytial Virus.

**Table S4: Clinical characteristics and healthcare resource utilization for adults hospitalized with RSV, influenza, acute MI, and stroke stratified by age group**

| **Characteristics** | **RSV** | | **Influenza** | | **Acute MI** | | **Stroke** | |
| --- | --- | --- | --- | --- | --- | --- | --- | --- |
|  | **18-64**  **[N = 1,070]** | **≥ 65**  **[N = 2,559]** | **18-64**  **[N = 128,751]** | **≥ 65**  **[N = 174,826]** | **18-64**  **[N = 180,234]** | **≥ 65**  **[N = 208,448]** | **18-64**  **[N = 142,648]** | **≥ 65**  **[N = 274,102]** |
| CCI  [Mean (SD)] | 2.7 (2.5) | 2.9  (2.3) | 2.5  (2.4) | 3.4  (2.5) | 2.5  (1.9) | 3.6  (2.3) | 3.5  (2.1) | 4.0  (2.3) |
| Risk conditions [N (%)] | | | | | | | | |
| Cardiopulmonary | 44 (4.1) | 154  (6.0) | 6,740 (5.2) | 14,539 (8.3) | 2,996 (1.7) | 8,578 (4.1) | 1,554 (1.1) | 6,938 (2.5) |
| Cardiovascular | 399 (37.3) | 1,631 (63.7) | 48,963 (38.0) | 117,586 (67.3) | 180,234 (100.0) | 208,448 (100.0) | 50,409 (35.3) | 153,140 (55.9) |
| Hematological | 226 (21.1) | 412 (16.1) | 15,798 (12.3) | 26,198 (15.0) | 9,502  (5.3) | 21,254 (10.2) | 7,742  (5.4) | 19,507  (7.1) |
| Hepatic | 49 (4.6) | 55  (2.1) | 7,967 (6.2) | 4,979 (2.8) | 3,238 (1.8) | 2,931 (1.4) | 3,469 (2.4) | 3,440 (1.3) |
| Metabolic | 386 (36.1) | 963 (37.6) | 41,415 (32.2) | 67,208 (38.4) | 67,410 (37.4) | 90,256 (43.3) | 59,321 (41.6) | 108,082 (39.4) |
| Neurologic | 143 (13.4) | 754 (29.5) | 18,136 (14.1) | 49,116 (28.1) | 33,992 (18.9) | 69,032 (33.1) | 125,257 (87.8) | 252,274 (92.0) |
| Pulmonary | 540 (50.5) | 1,051 (41.1) | 90,156 (70.0) | 125,915 (72.0) | 32,621 (18.1) | 50,363 (24.2) | 23,776 (16.7) | 55,564 (20.3) |
| Renal | 244 (22.8) | 823 (32.2) | 20,923 (16.3) | 53,398 (30.5) | 23,310 (12.9) | 64,465 (30.9) | 19,313 (13.5) | 62,658 (22.9) |
| Immuno-compromising | 287 (26.8) | 334 (13.1) | 18,229 (14.2) | 27,549 (15.8) | 7,797  (4.3) | 15,010  (7.2) | 9,230  (6.5) | 21,301  (7.8) |
| Obesity-related | 219 (20.5) | 205  (8.0) | 27,205 (21.1) | 18,475 (10.6) | 21,927 (12.2) | 12,662  (6.1) | 16,276 (11.4) | 12,225  (4.5) |
| Risk Group [N (%)] | | | | | | | | |
| High | 962 (89.9) | 2,349 (91.8) | 113,927 (88.5) | 168,112 (96.2) | 180,234 (100.0) | 208,448 (100.0) | 134,845 (94.5) | 267,414 (97.6) |
| Healthcare Resource Utilization | | | | | | | | |
| ICU admission [N (%)] | 35  (3.3) | 76  (3.0) | 28,964 (22.5) | 29,438 (16.8) | 55,309 (30.7) | 50,982 (24.5) | 37,578 (26.3) | 56,060 (20.5) |
| MV Use [N (%)] | 10  (0.9) | 9  (0.4) | 24,138 (18.7) | 23,960 (13.7) | 10,755  (6.0) | 12,357  (5.9) | 2,705  (1.9) | 3,822  (1.4) |
| Length of inpatient stay [Mean (SD)] | 3.9 (3.1) | 4.0  (2.6) | 4.2  (4.9) | 4.4  (4.1) | 3.0  (2.7) | 3.7  (3.3) | 4.4  (6.1) | 4.1  (4.0) |
| Length of ICU stay [Mean (SD)] | 2.3 (2.8) | 2.3  (2.1) | 2.8  (3.9) | 2.8  (3.6) | 1.4  (1.4) | 1.7  (1.8) | 2.2  (2.9) | 1.7  (1.9) |

List of Abbreviations: CCI: Charlson Comorbidity Index; ICU: Intensive Care Unit; MI: Myocardial Infarction; MV: Mechanical Ventilator; N: Number of unique hospitalizations; RSV: Respiratory Syncytial Virus.
